# Coherent Target Cancellation and Deceptive Jamming Through a Multi-Region Time-Coding Metasurface

**DOI:** 10.3390/s26175474

**Published:** 2026-08-29

**Authors:** Chen Zhang, Wenjuan Qi, Xu Qi, Zhifeng Cheng, Xuekai Lan, Lirui Xu, Diwei Liu

**Affiliations:** 1 Naval University of Engineering, Wuhan 430033, China; 2320232070@nue.edu.cn (C.Z.); 1920191205@nue.edu.cn (W.Q.); 2320236081@nue.edu.cn (X.L.); 2420241049@nue.edu.cn (L.X.); 2 Terahertz Science and Technology Research Center, University of Electronic Science and Technology of China, Chengdu 610054, China; dwliu@uestc.edu.cn

**Keywords:** radar countermeasures, coherent cancellation, deceptive jamming, multi-region time-coding metasurface (MRTCM)

## Abstract

**Highlights:**

**What are the main findings?**

- Cooperative synthesis by distributed time-coding metasurface regions suppresses the true target by more than 40 dB in simulated high-resolution range profiles while preserving controllable harmonic false targets.
- Zeroth-order cancellation is invariant to cyclic coding-sequence shifts and remains effective during asynchronous multi-pulse processing, whereas harmonic-based cancellation requires accurate timing and synchronization.

**What is the implication of the main finding?**

- Scattering centers that cannot be directly covered by a metasurface may still be protected using coherently controlled metasurface regions installed on other available platform surfaces.

**Abstract:**

Existing metasurface-based radar countermeasures commonly manipulate echoes from metasurface-covered regions, whereas protecting exposed scattering centers that cannot accommodate metasurfaces remains challenging. This paper presents a multi-region time-coding metasurface (MRTCM) architecture for coherent target cancellation and deceptive jamming. The proposed system uses multiple independently controlled metasurface regions distributed over available platform surfaces. Each region generates zeroth-order and higher-order harmonic components through periodic phase modulation, and these components coherently combine with echoes from uncovered areas. The zeroth-order component is invariant under cyclic shifts of the coding sequence, enabling stable cancellation during multi-pulse coherent processing without locking radar-pulse arrival to the start of the coding cycle. Higher-order harmonics can shift the apparent range of the metasurface response and support cancellation at range-separated cells, but require accurate timing and synchronization. Numerical simulations demonstrate more than 40 dB suppression in high-resolution range profiles and validate the cancellation behavior under moving-target detection processing. The MRTCM architecture therefore provides a theoretically grounded framework for electromagnetic protection when full metasurface coverage is impractical.

## 1. Introduction

Electronic warfare (EW) has become a critical domain in modern military operations, where controlling electromagnetic signatures determines platform survivability [1]. Traditional jamming techniques face limitations in countering modern radar systems. Active jamming reveals platform position through high-power emissions and increases vulnerability to anti-radiation missiles [2]. Passive techniques lack dynamic adaptability and cannot respond to real-time parameter changes [3]. These challenges intensify against modern radars employing frequency agility, polarization diversity, and cognitive processing algorithms [4].

Metasurfaces offer exceptional electromagnetic wave manipulation capabilities, presenting significant potential for radar stealth and jamming applications [5,6]. Multi-faced transmissive metadevices have also demonstrated coordinated wavefront and polarization functions across several controlled facets [7].

In stealth applications, metasurfaces achieve radar cross-section (RCS) reduction through various mechanisms. Checkerboard structures utilizing 180° phase differences between adjacent cells demonstrate 10 dB reduction across 5.5–32.3 GHz [8], while gradient metasurfaces redirect incident waves through spatially varying phase distributions [9]. Polarization conversion structures maintain cross-polarization ratios above 0.9 even at oblique incidence angles up to 50° [10]. More sophisticated designs integrate multiple techniques: coding metasurfaces employ optimized discrete phase sequences for programmable wave control [5], absorptive structures achieve 90% absorption from 2.2 to 18 GHz [11], and recent absorptive coding metasurfaces combine absorption with diffusion mechanisms, extending operational bandwidth to 7.5–45.2 GHz [12]. Programmable coding metasurfaces have likewise demonstrated independent dynamic control of absorption and scattering [13].

Beyond passive RCS reduction, time-modulated metasurfaces enable active radar countermeasures through spatiotemporal control of reflection coefficients. For deception jamming, external time-coding metasurfaces modulate target echoes to achieve enhancement or suppression without physical attachment [14]. Diverse frequency–time modulation using independently controlled elements generates multiple false targets with controllable positions in high-resolution range profiles (HRRPs) [15]. Velocity deception is achieved by temporally modulating metasurface resonances to imprint artificial Doppler signatures, shifting perceived target speeds to arbitrary values [16,17]. Time-domain digital coding enables micro-Doppler signature simulation without mechanical movement [18]. For imaging radars, information metasurfaces disrupt synthetic aperture radar (SAR) through rapid backscattering coefficient modulation [19], while inverse synthetic aperture radar (ISAR) is countered via time-modulated phases that blur or duplicate target images [20]. Cross-eye jamming against monopulse radars employs 1-bit coding metasurfaces generating coherent signals with 180° phase difference [21].

In suppression jamming, random phase modulation disperses spectral energy with effectiveness increasing as switching intervals decrease [22], while time-varying metasurfaces create broadband noise-like interference through programmable voltage control [23,24]. Space-time-coding metasurfaces counter multi-static radar by generating different harmonics at each receiver, defeating localization algorithms [25]. These techniques achieve comparable jamming performance to traditional systems with reduced hardware complexity and power consumption through passive or semi-passive operation.

**Figure 1:**
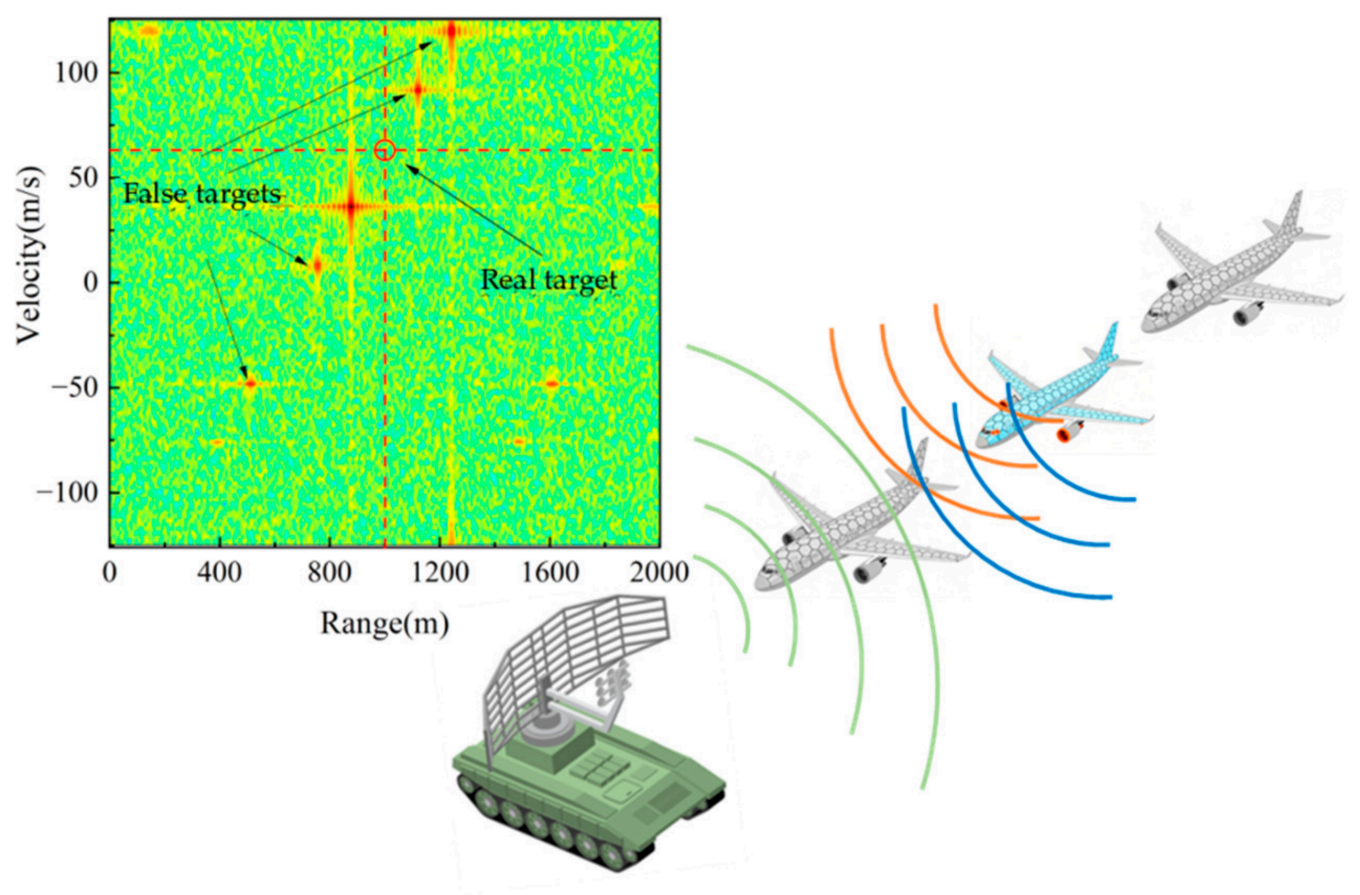
Schematic deployment of the multi-region time-coding metasurface (MRTCM). Time-coding metasurfaces on available aircraft surfaces generate zeroth-order and higher-order harmonic components that coherently cancel echoes from exposed cockpit and engine regions, leaving harmonic false-target signatures in the composite radar return. The green arcs denote incident radar waves; the blue arcs denote metasurface-modulated scattering; and the orange arcs denote scattering from the exposed cockpit and engine. In the range–velocity map, the red dashed horizontal and vertical lines denote the true-target velocity and range references, respectively; the red circle denotes the true target position; and the black arrows identify the true and false response peaks.

Although external time-coding metasurfaces can modulate the high-resolution range profile (HRRP) of a spatially separated target [14], cooperative amplitude–phase synthesis among multiple distributed metasurface regions and its temporal stability under multi-pulse coherent processing remain insufficiently investigated. To address this gap, this paper proposes a multi-region time-coding metasurface (MRTCM) architecture for protecting scattering centers that cannot be directly covered by metasurfaces. The MRTCM comprises multiple independently controlled regions distributed over available platform surfaces. Each region generates zeroth-order and higher-order harmonic components through time modulation, and these components coherently superpose with echoes from uncovered areas. By exploiting the coherent interaction between metasurface-generated signals and target reflections, the composite echo suppresses the true target signature while retaining harmonic false-target components, producing a “conceal-the-real, reveal-the-false” effect, as illustrated in Figure 1.

The proposed MRTCM architecture supports two operating scenarios. First, for co-range protection, multiple metasurface regions located in the same range cell as critical uncovered components use their zeroth-order components for target cancellation. This configuration is applicable to exposed engine intakes, rotating machinery, sensor apertures that cannot accommodate surface coatings, and other dominant scattering centers within the same range cell; cancellation is examined under both single-pulse and multi-pulse processing. Second, for range-separated protection, metasurface regions located at different ranges use higher-order harmonics with controllable range offsets to align with and cancel echoes from spatially separated targets. This mode requires accurate time-of-arrival information and synchronization, but offers greater flexibility in metasurface placement.

The remainder of this paper is organized as follows. Section 2 formulates the theoretical MRTCM scattering model and derives the pulse-compression response under linear frequency-modulated (LFM) illumination. Section 3 analyzes the two cancellation mechanisms: zeroth-order-component-based co-range cancellation and harmonic-based range-separated cancellation. Section 4 presents numerical validation using wideband HRRP analysis and narrowband moving-target detection (MTD) processing. Section 5 concludes the paper and discusses the principal limitation.

## 2. Theoretical Model and Signal Analysis

Radar systems detect targets by analyzing scattered electromagnetic waves and extracting information from echo characteristics including time delay, amplitude, and phase. The MRTCM generates controllable zeroth-order and higher-order harmonic components through time-varying phase modulation of multiple independent regions. Coherent combination of these spectral components enables precise tailoring of the composite scattering response to achieve desired electromagnetic signatures.

### 2.1. Scattering Model of Multi-Region Time-Coding Metasurface

**Figure 2:**
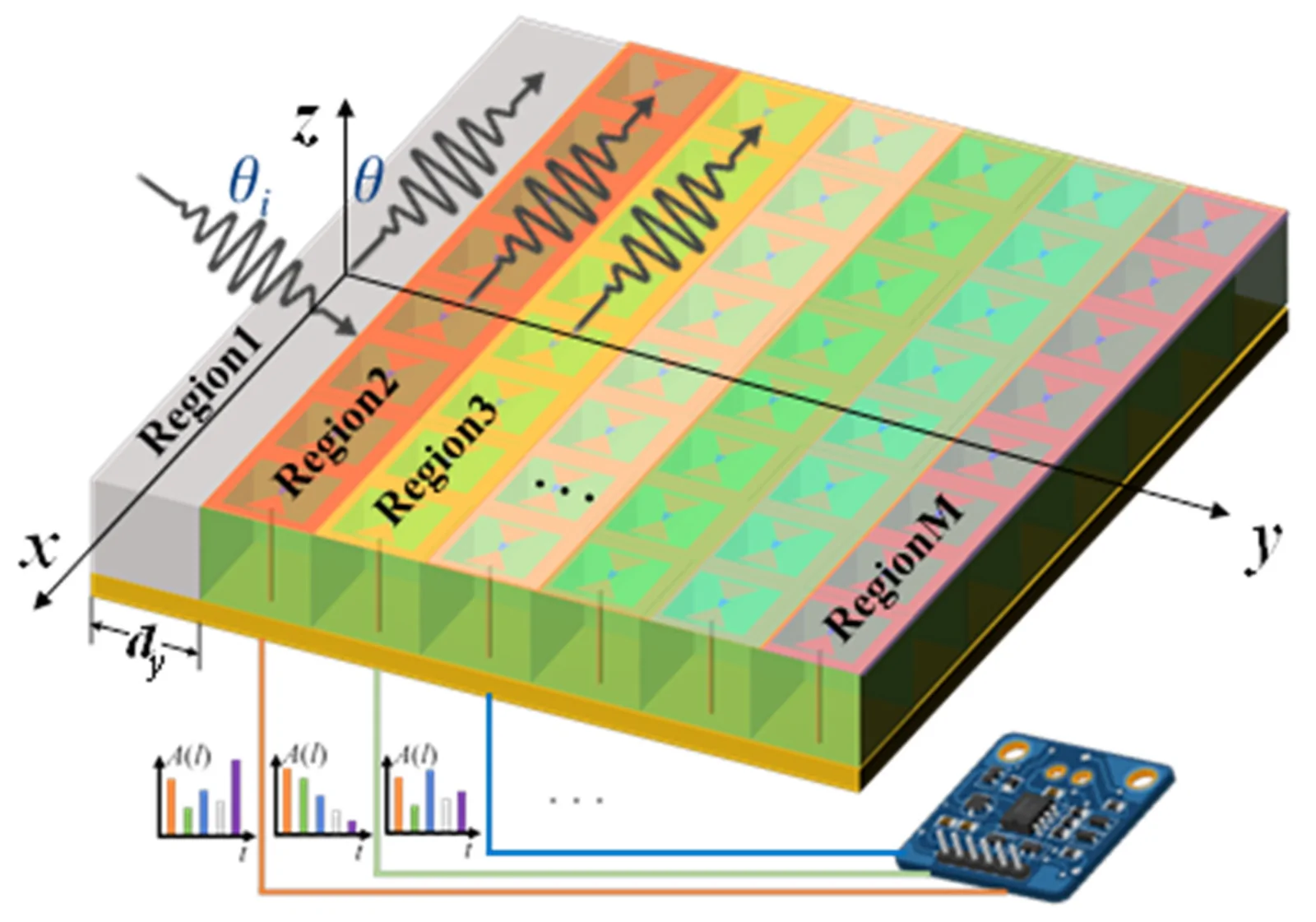
Geometry of the multi-region time-coding metasurface. The incidence angle $\theta_{i}$ and observation angle θ are measured from the surface normal in the y − z plane, and the center of region m is located at $y_{m}=\left(m-1\right)d_{y}$. Different colors distinguish the individual metasurface regions, and the ellipsis denotes intermediate regions omitted for clarity.

Consider an MRTCM composed of M independently controlled regions arranged along the y-direction, as illustrated in Figure 2. The center coordinate of the m th region is denoted by $y_{m}=\left(m-1\right)d_{y}$, m=1,2,…,M, where $d_{y}$ is the center-to-center spacing between adjacent regions. Each region is controlled by an independent periodic coding function $p_{m}\left(\tau \right)$. Mutual coupling between regions is neglected, and the incident and scattered fields are assumed to satisfy the far-field approximation.

With $D_{tot}$ denoting the overall aperture size, the far-field formulation is used when the relevant propagation distances are substantially larger than $2D_{tot}^{2}/\lambda$. At shorter distances, the plane-wave spatial phases in Equations (1)–(3) should be replaced by spherical-wave propagation terms. If inter-region coupling is appreciable, the independent regional factors must instead be obtained jointly from full-wave simulation or measurement; a quantitative coupling model is left for future work.

Under the $e^{j2\pi f\tau }$ time convention, the field scattered toward the observation direction θ is obtained by coherently summing the contribution of each region:

(1) $$r(\theta ,\tau )=s(\tau )\sum_{m=1}^{M}G_{m}(\theta_{i},k_{c})H_{m}(\theta ,k_{o},\tau )$$

where $s\left(\tau \right)$ is the incident radar signal, $\theta_{i}$ is the incidence angle, θ is the observation angle, $k_{c}=2\pi f_{c}/c$ is the incident wavenumber at the carrier frequency, and $k_{o}$ is the wavenumber of the scattered component. Equation (1) combines the illumination, temporal modulation, and outgoing radiation of each physical region before coherent summation. This region-wise formulation preserves the phase relationships among independently modulated regions.

The incident-field coupling coefficient of the m th region is expressed as

(2) $$G_{m}\left(\theta_{i},k_{c}\right)=F_{m}^{\mathrm{in}}\left(\theta_{i},k_{c}\right)\exp\left(-jk_{c}y_{m}\sin\theta_{i}\right),$$

where $F_{m}^{in}\left(\theta_{i},k_{c}\right)$ represents the complex receiving or illumination factor of the m th region. It may include the incident-field amplitude, polarization response, regional pattern, and any region-dependent static phase offset.

The time-varying scattering response of the same region is written as

(3) $$H_{m}\left(\theta ,k_{o},\tau \right)=\sigma_{m}F_{m}^{\mathrm{out}}\left(\theta ,k_{o}\right)p_{m}\left(\tau \right)\exp\left(jk_{o}y_{m}\sin\theta \right),$$

where $\sigma_{m}$ is the effective scattering-amplitude coefficient of the m th region and $F_{m}^{out}\left(\theta ,k_{o}\right)$ is its complex radiation factor in the observation direction. Here, $\sigma_{m}$ denotes a field-amplitude coefficient rather than scattered power; the associated power or RCS contribution is proportional to ${\left|\sigma_{m}\right|}^{2}$. Combining Equations (1)–(3), the contribution of the m th region contains the spatial phase factor $exp\left(j\left[k_{o}sin\theta -k_{c}sin\theta_{i}\right]y_{m}\right)$. The angles follow the coordinate convention in Figure 2; in a monostatic backscatter geometry, the incidence and observation directions are linked by the backscatter condition.

Within one modulation period, the coding function of the m th region is represented by a sequence of $L_{m}$ rectangular chips:

(4) $$\begin{array}{l} p_{m}(\tau )=\sum_{l=1}^{L_{m}}A_{m}(l)\cdot \mathrm{rect}\left(\frac{\tau -(l-1/2)T_{0,m}}{T_{0,m}}\right),\;0\leq \tau <T_{m},\\ p_{m}\left(\tau +T_{m}\right)=p_{m}\left(\tau \right). \end{array}$$

Here, $T_{m}=L_{m}T_{0,m}$ is the modulation period and $T_{0,m}$ is the chip duration. We define $rect\left(x\right)=1$ for $\left|x\right|<1/2$, $rect\left(x\right)=1/2$ for $\left|x\right|=1/2$, and $rect\left(x\right)=0$ for $\left|x\right|>1/2$; the endpoint convention does not affect the Fourier coefficients. For the 2-bit phase-only states, $A_{m}\left(l\right)\in \{1,j,-1,-j\}$. In Table 1 and Table 2, the integer labels 1, 2, 3, and 4 denote 1, j, −1, and −j, respectively.

Because $p_{m}\left(\tau \right)$ is periodic, it can be expanded into a Fourier series:

(5) $$p_{m}(\tau )=\sum_{u=-\infty }^{+\infty }\alpha_{u,m}\exp\left(j2\pi uf_{s,m}\tau \right),\;f_{s,m}=\frac{1}{T_{m}},$$

where u is the harmonic order and $\alpha_{u,m}$ is the complex Fourier coefficient of the u th harmonic. When all regions use a common modulation period, $f_{s,m}=f_{s}$ for every m, although their sequence lengths and chip durations may differ.

Using Equation (4), the Fourier coefficient is obtained as

(6) $$\begin{array}{cc} \alpha_{u,m} & =\frac{1}{T_{m}}\int_{0}^{T_{m}}p_{m}(\tau )\exp\left(-j2\pi uf_{s,m}\tau \right)d\tau \\ & =\frac{1}{L_{m}}\sin c\left(\frac{u}{L_{m}}\right)\sum_{l=1}^{L_{m}}A_{m}(l)\exp\left[-\frac{j\pi u(2l-1)}{L_{m}}\right], \end{array}$$

where $sinc\left(x\right)=sin\left(\pi x\right)/\left(\pi x\right),\;sinc\left(0\right)=1$. For the zeroth-order component, Equation (6) reduces to $\alpha_{0,m}=\frac{1}{L_{m}}\sum_{l=1}^{L_{m}}A_{m}\left(l\right)$. Therefore, $\alpha_{0,m}$ depends only on the number of occurrences of each coding state and is independent of their temporal ordering. By contrast, the higher-order coefficients $\alpha_{u,m}$, u≠0, depend on both the coding states and their positions within the sequence. This distinction provides the theoretical basis for timing-robust zeroth-order cancellation and timing-sensitive harmonic manipulation discussed below.

### 2.2. Pulse-Compression Response Under LFM Illumination

To analyze the system response to practical radar waveforms, consider a pulsed radar system transmitting a linear frequency-modulated (LFM) chirp signal:

(7) $$s(\tau )=\mathrm{rect}\left(\frac{\tau }{T_{p}}\right)\exp\left(j\pi K_{r}\;\tau^{2}\right)\exp\left(j2\pi f_{c\;}\tau \right),$$

where $rect\left(\tau /T_{p}\right)$ denotes the rectangular pulse envelope of duration $T_{p}$, and $K_{r}=B/T_{p}$ represents the frequency sweep rate for bandwidth B.

For simplicity in the subsequent analysis, the target is assumed to exhibit isotropic scattering with a constant complex field-scattering coefficient over the operational bandwidth and angular range. For a point target at range $R_{i}$ with field-amplitude coefficient $\sigma_{i}$ and intrinsic scattering phase $\psi_{i}$, the received echo signal is:

(8) $$r_{i}\;(\tau )=\sigma_{i\;}\mathrm{rect}\left(\frac{\tau -\tau_{i}}{T_{p}}\right)\exp\left[j\pi K_{r}\;{\left(\tau -\tau_{i}\right)}^{2}\right]\exp\left[j2\pi f_{c}\;(\tau -\tau_{i})\right]\exp\left(j\psi_{i}\right)\;$$

where $\tau_{i}=2R_{i}/c$ is the round-trip time delay.

After pulse compression using matched filtering, the compressed output becomes:

(9) $$\gamma_{i}\;(\tau )\;=\;\sigma_{i}T_{p}\left(1-\frac{\left|\tau -\tau_{i}\right|}{T_{p}}\right)\exp\left(-j2\pi f_{c}\tau_{i}+j\psi_{i}\right)\sin c\left[B(\tau -\tau_{i})\left(1-\frac{\left|\tau -\tau_{i}\right|}{T_{p}}\right)\right]$$

The above analysis applies to point targets. For the MRTCM system under normal incidence ($\theta_{i}=0$), the composite field at observation angle θ is obtained by coherently combining the M regional contributions. In the numerical examples, the calibrated regional product $F_{m}^{in}\left(\theta_{i},k_{c}\right)F_{m}^{out}\left(\theta ,k_{o}\right)$ is normalized to unity; with $k_{o}≃k_{c}$, the m th regional contribution is given by Equation (10).

(10) $$r_{m}(\tau )=\sigma_{m}s(\tau -\tau_{m})p_{m}(\tau -\frac{\tau_{m}}{2})\exp\left[jk_{o}\left(m-1\right)d_{y}sin\theta \right]$$

where $\tau_{m}=2R_{m}/c$ represents the round-trip delay to the metasurface located at range $R_{m}$, $p_{m}\left(\tau -\tau_{m}/2\right)$ is the time-varying modulation applied when the signal reaches the metasurface. Since the carrier frequency is much larger than the modulation frequency ($f_{c}\gg f_{s}$), the wave number of the outgoing wave $k_{o}\approx k_{c}$ for all harmonics.

Substituting the Fourier expansion of $p_{m}$(τ), the pulse-compression output from the m th region yields:

(11) $$\begin{array}{cc} \gamma_{m}(\tau ) & =\sigma_{m}T_{p}\sum_{u=-\infty }^{\infty }\alpha_{u,m}\left(1-\frac{\left|\tau -\tau_{m}\right|}{T_{p}}\right)\exp\left[j2\pi \left(\frac{uf_{s}\tau }{2}-f_{c}\tau_{m}\right)\right]\\ & \cdot \exp\left[jk_{c}\left(m-1\right)dysin\theta \right]\sin c\left[B\left(\tau -\tau_{m}+\frac{uf_{s}}{K_{r}}\right)\left(1-\frac{\left|\tau -\tau_{m}\right|}{T_{p}}\right)\right] \end{array}$$

Equation (11) reveals that the u-th harmonic component appears at the range position $\tau =\tau_{m}-uf_{s}/K_{r}$. Its complex amplitude is determined by the corresponding Fourier coefficient and harmonic order. Because the harmonic coefficients can be tailored through coding-sequence design and the apparent range positions can be set through the modulation frequency, the pulse-compression response can be engineered for coherent cancellation and deceptive false-target generation.

## 3. Multi-Region Cooperative Cancellation and Deception Mechanism

Finite reflection states produce discrete zeroth-order and higher-order coefficients in the amplitude–phase plane. Increasing the sequence length refines the available coefficient set, but a single region generally cannot independently match an arbitrary target amplitude and phase. Multi-region coherent synthesis addresses this limitation by combining several independently programmable complex contributions. For a fixed modulation period, however, longer sequences also require shorter chip durations and therefore higher switching rates.

### 3.1. Co-Range Target Cancellation via the Zeroth-Order Component

For notational convenience, the uncovered scattering contribution to be cancelled is indexed by m=1, although it is not itself a metasurface region. It has field-amplitude coefficient $\sigma_{1}$ and intrinsic scattering phase $\psi_{1}$, whereas regions m=2,3,…,M are independently coded cancellation regions.

According to Equation (9), the peak amplitude and phase of the uncovered contribution are given by Equation (12), where $arg\left(z\right)$ denotes the principal argument of a nonzero complex number.

(12) $$\begin{array}{c} \left|\left(\gamma_{1}(\tau_{1}\right)\right|=\sigma_{1}T_{p}\\ arg\left[\left(\gamma_{1}(\tau_{1}\right)\right]=-2\pi f_{c}\tau_{1}+\psi_{1} \end{array}$$

For the modulated regions (m=2,3,…,M), define the peak location of harmonic order u as $\tau_{m,u}=\tau_{m}-uf_{s}/K_{r}$. Its peak magnitude and phase are given by Equation (13).

(13) $$\begin{array}{cc} \tau_{m,u} & =\tau_{m}-\frac{uf_{s}}{K_{r}},\\ \left|\gamma_{m,u}(\tau_{m,u})\right| & =\sigma_{m}T_{p}\left(1-\frac{\left|uf_{s}\right|}{B}\right)\left|\alpha_{u,m}\right|,\\ \arg[\gamma_{m,u}(\tau_{m,u})] & =2\pi \left[\frac{uf_{s}\tau_{m}}{2}-\frac{{\left(uf_{s}\right)}^{2}}{2K_{r}}-f_{c}\tau_{m}\right]+k_{c}\left(m-1\right)d_{y}\sin\theta +\arg\left(\alpha_{u,m}\right),\\ \left|uf_{s}\right| & <B. \end{array}$$

In the ideal equal-delay co-range case, $\tau_{1}=\tau_{2}=\cdots =\tau_{M}$, cancellation is performed using the zeroth-order component, u=0.

**Figure 3:**
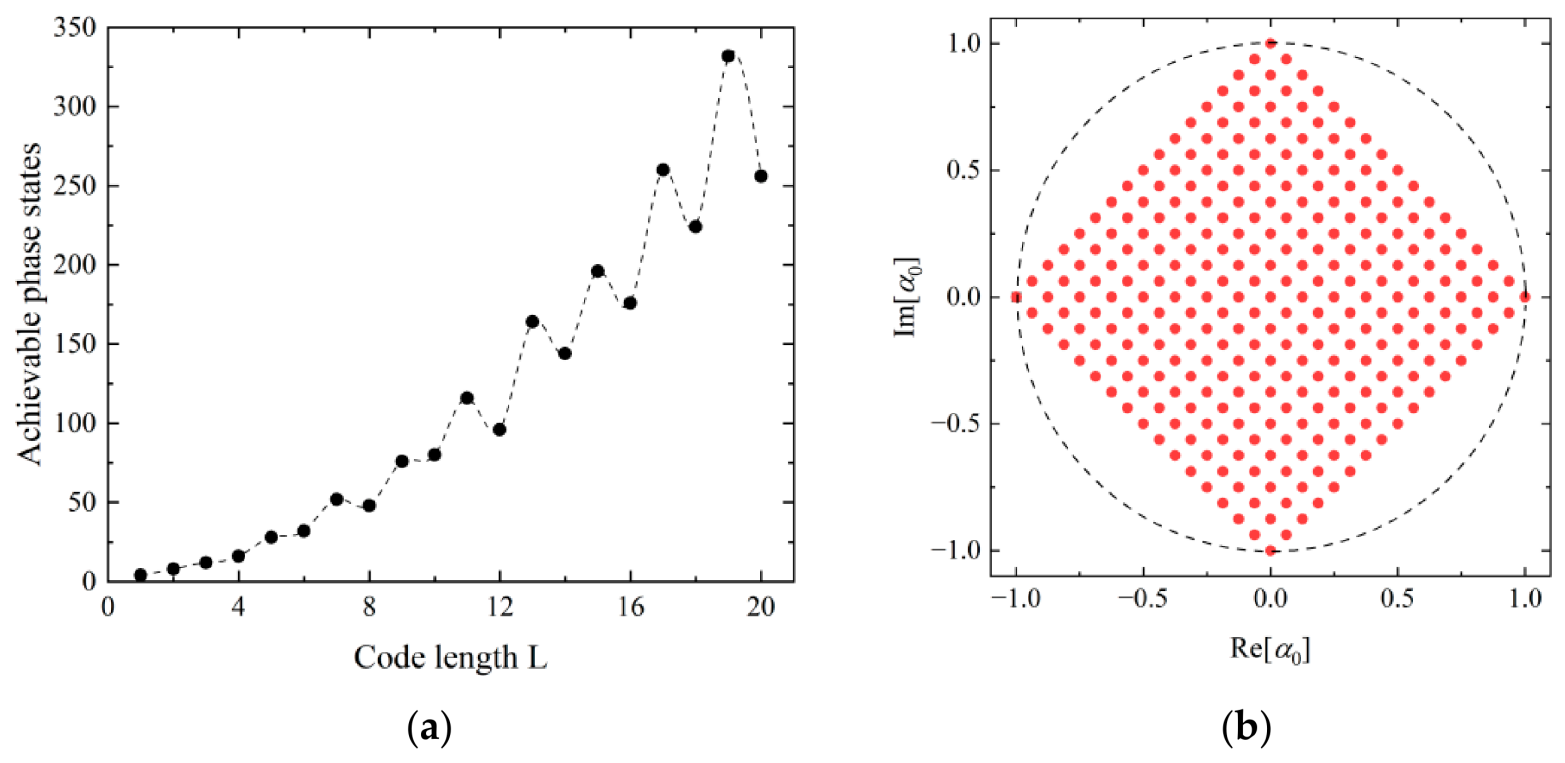
Analysis of phase control for 2-bit encoding. (**a**) Number of distinct nonzero phases versus code length $L_{m}$. (**b**) Distribution of $\alpha_{0}$ in the complex plane for $L_{m}=16$. The dotted circle in (**b**) denotes the unit circle |α_0_| = 1.

For 1-bit phase encoding where $A\left(l\right)\in \{+1,-1\}$, the zeroth-order Fourier coefficient $\alpha_{0}$ yields only phases of 0 or π. This binary phase limitation prevents cancellation of targets with arbitrary scattering phases $\psi_{1}\notin \{0,\pi \}$. While 2-bit encoding expands the achievable phase states, the discrete nature still limits flexibility. Figure 3a shows the relationship between sequence length and achievable phase states for 2-bit encoding, while Figure 3b illustrates the Fourier coefficient for a code length of $L_{m}=16$.

The oscillation in Figure 3a follows from the discrete four-state alphabet. If $N_{1},N_{2},N_{3},N_{4}$ are the numbers of 1,j,−1,−j, respectively, then $\alpha_{0}=\left(a+jb\right)/L$, where $a=N_{1}-N_{3}$, $b=N_{2}-N_{4}$, $\left|a\right|+\left|b\right|\leq L$, and $L-\left|a\right|-\left|b\right|$ is even. Adjacent odd and even lengths therefore occupy different parity sublattices, while nonzero points on the same oriented ray have the same phase. The zero coefficient is excluded because its phase is undefined.

At the protected range sample, the discrete complex contributions predicted by Equation (13) are combined coherently. For region m, the vector magnitude is determined by $\sigma_{m}\left|\alpha_{u,m}\right|$ and the pulse-compression factor, while its phase is determined by $arg\left(\alpha_{u,m}\right)$, the propagation delay, and the spatial phase. The regional sequences are chosen jointly from the available 2-bit states so that the vector sum approaches the negative of the target response.

Specifically, using regions 2 and 3 for cancelling region 1, the condition becomes:

(14) $$\sigma_{1}e^{j\psi_{1}}+\sigma_{2}\alpha_{0,2}e^{jk_{c}dysin\theta }+\sigma_{3}\alpha_{0,3}e^{j2k_{c}dysin\theta }=0$$

Equation (14) describes the ideal equal-delay case. In practice, compressed echoes occupy the same range cell when their envelopes overlap, approximately $\left|\Delta \tau \right|≲1/B$ or $\left|\Delta R\right|≲c/\left(2B\right)$. With unequal delays, cancellation at the protected sample is evaluated from the full complex responses in Equations (9) and (11), which retain the envelope and carrier-phase differences.

Crucially, the zeroth-order Fourier coefficient depends only on the number of occurrences of each coding state and not on their temporal order. This exact invariance to cyclic sequence shifts provides strong timing robustness in multi-pulse radar scenarios.

For a code-clock offset $\Delta t_{c}$, defined by ${\tilde{p}}_{m}\left(t\right)=p_{m}\left(t-\Delta t_{c}\right)$, the Fourier shift theorem gives ${\tilde{\alpha }}_{u,m}=\alpha_{u,m}e^{-j2\pi uf_{s}\Delta t_{c}}$. Hence ${\tilde{\alpha }}_{0,m}=\alpha_{0,m}$: pulse arrival need not coincide with the start of the coding cycle for zeroth-order cancellation. This invariance concerns code phase only and does not relax the requirements on range overlap or carrier-phase coherence.

### 3.2. Range-Separated Target Cancellation via Harmonic Components

Higher-order harmonic components (u≠0) create apparent scattering responses at shifted range positions. The u-th harmonic appears at the range:

(15) $$R_{u}=-\frac{c\cdot u\cdot f_{s}}{2K_{r}}+R_{m}$$

For range-separated targets, the modulation frequency $f_{s}$ can be selected so that the u-th harmonic aligns with a target at range $R_{i}$, enabling coherent cancellation despite physical range separation.

The cancellation condition requires complex-amplitude matching between the target echo and the selected harmonic component. Although the principle is the same as in co-range cancellation, the higher-order coefficient $\alpha_{u}$ (u≠0) acquires an order-dependent phase under a cyclic sequence shift and is therefore more sensitive to timing.

For u≠0, the same code-clock offset leaves $\left|\alpha_{u,m}\right|$ and the nominal harmonic range unchanged but adds the phase $-2\pi uf_{s}\Delta t_{c}$. The harmonic peak therefore remains, although its phase no longer matches the target unless the code offset is known. A time-of-arrival (TOA) error caused by a propagation-delay error is different because it also changes the compressed-envelope position and carrier phase.

Across pulses, let PRT denote the pulse repetition time and PRF=1/PRT the pulse repetition frequency. Harmonic order u recurs when $uf_{s}/PRF\in Z$. Because $T_{m}=1/f_{s}$, $PRT/T_{m}=f_{s}/PRF$; hence $f_{s}/PRF\in Z$ guarantees recurrence for every integer harmonic order.

Coherent cancellation through a pulse train also requires the selected harmonic contribution to follow the target slow-time phase over the coherent processing interval.

## 4. Numerical Validation and Performance Analysis

To validate the theoretical framework established in Section 2 and Section 3, numerical simulations were conducted using an MRTCM configuration. The system comprises an uncovered target contribution indexed as region 1 for bookkeeping, and multiple actively modulated regions (regions 2 and 3) implementing 2-bit phase modulation for cooperative cancellation. Two operational scenarios are investigated: zeroth-order-component-based cancellation for co-range targets where all regions reside in the same range cell, and harmonic component-based manipulation for range-separated targets utilizing controllable range shifts. Furthermore, the analysis encompasses both wideband linear frequency modulation (LFM) scenarios examining HRRP, and narrowband configurations evaluating multi-pulse coherent processing performance under MTD conditions.

### 4.1. Zeroth-Order Component Performance in HRRP and MTD Processing

Table 1 summarizes the system parameters for zeroth-order-component-based co-range cancellation. For this analysis, the observation angle is set to θ = 37°, with all three regions (target and modulation regions) positioned at $R_{1}$ = $R_{2}$ = $R_{3}$ = 1000 m. The element spacing along the y-direction is $d_{y}$ = 15 mm. The wideband configuration employs B = 500 MHz bandwidth for complex HRRP generation.

**Figure 4:**
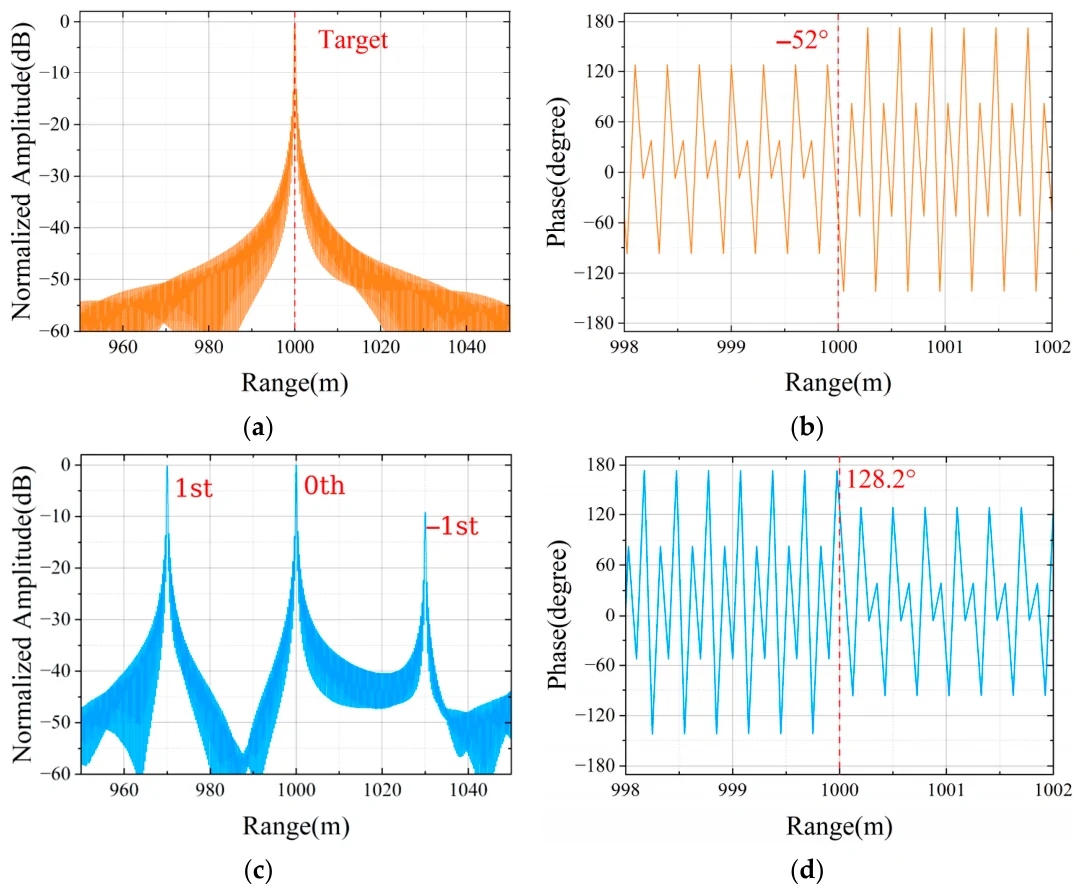

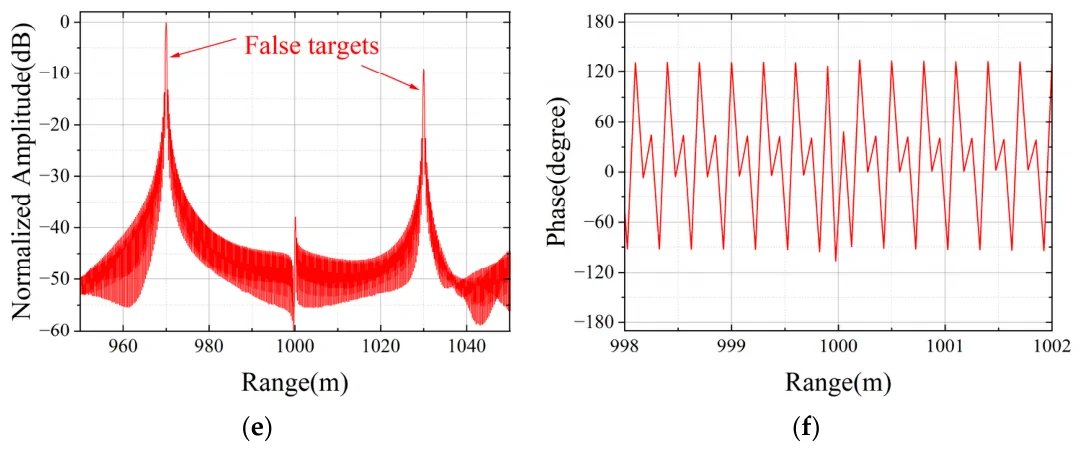
Complex high-resolution range profile (HRRP) of zeroth-order-component-based co-range cancellation. (**a**) Target amplitude. (**b**) Target phase. (**c**) Modulated metasurface amplitude. (**d**) Modulated metasurface phase. (**e**) Synthesized amplitude. (**f**) Synthesized phase.

Figure 4 presents the complex HRRP cancellation mechanism. For phase display, all complex responses are referenced to the incident field at the target-range plane $R_{ref}=R_{1}=1000$ m, such that $\gamma_{fig}\left(\tau \right)=\gamma \left(\tau \right)e^{jk_{c}R_{ref}}$. Under this common reference, the target vector is 1∠(−52°) and regions 2 and 3 synthesize approximately 1.00865∠128.222°. The common reference transformation changes neither their relative phase (approximately 180°) nor the cancellation depth.

The synthesized HRRP in Figure 4e,f combines all three contributions. At 1000 m, the residual amplitude is suppressed below −40 dB, while the time-coding harmonics remain visible at other range cells. At an exact complex zero the phase is undefined, and near the finite numerical null it is ill-conditioned; the phase curve in Figure 4f should therefore be interpreted only away from the cancellation point.

In practical applications, exact target parameters and observation angles may not be fully available. The system must therefore tolerate parameter uncertainties. The cancellation performance is analyzed under two mismatch scenarios: scattering phase deviation and observation angle variation.

**Figure 5:**
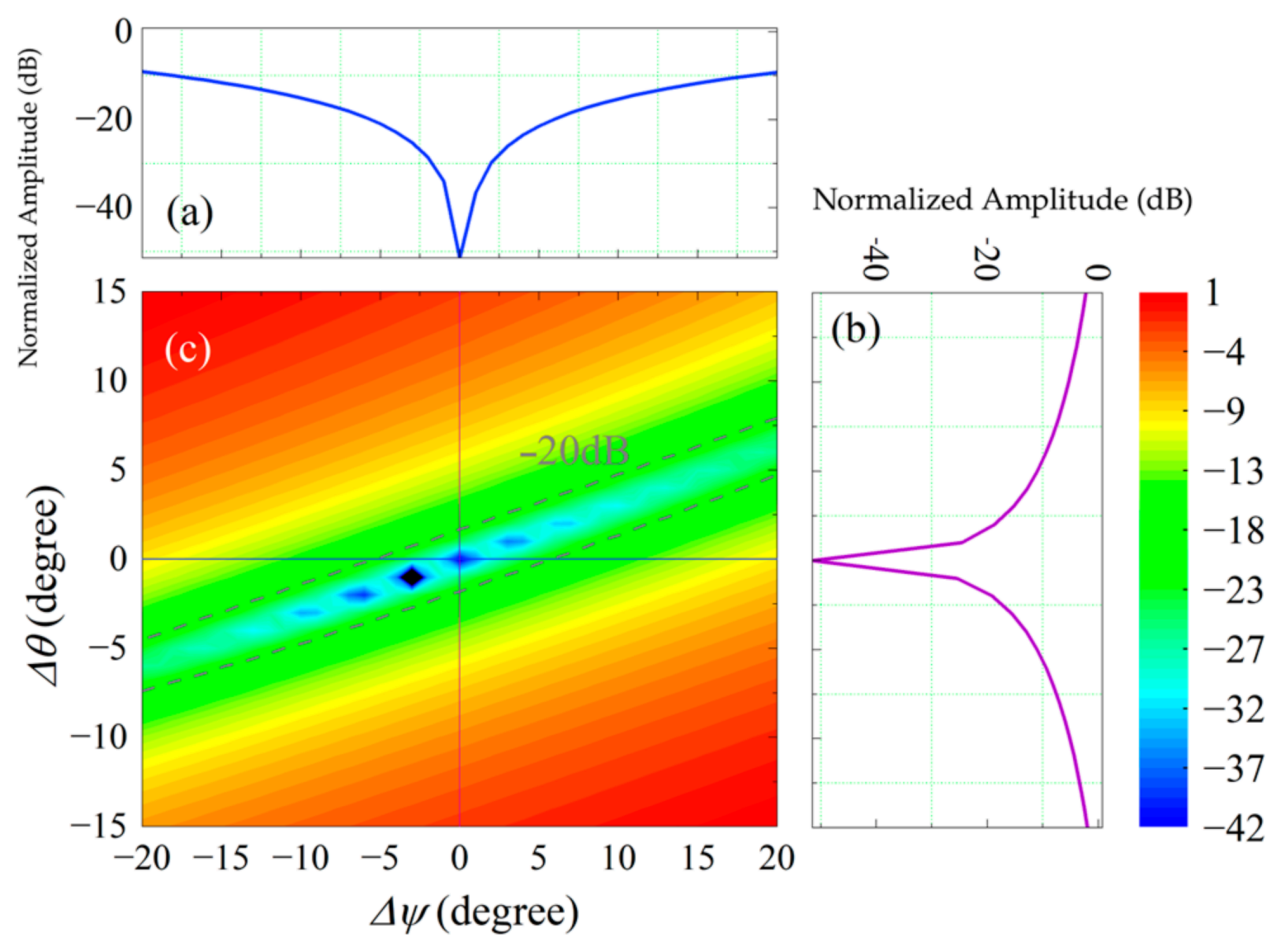
Cancellation-performance sensitivity analysis. (**a**) Normalized amplitude versus target phase deviation Δψ. (**b**) Normalized amplitude versus observation-angle deviation Δθ. (**c**) Two-dimensional contour map of cancellation depth in the (Δψ, Δθ) parameter space. The blue horizontal and vertical lines denote the zero-deviation cuts, and the purple dashed contours denote the −20 dB level.

For phase mismatch analysis, the observation angle is fixed at θ = 37° while the actual target phase varies within [48°, 88°] around the 68° design value. Figure 5a illustrates the normalized amplitude variation with phase deviation Δψ, showing acceptable performance (below −20 dB) within approximately ±6° deviation.

For angular sensitivity analysis, the target phase remains at $\psi_{1}=$ 68° while the observation angle varies from 22° to 52°. Figure 5b gives an angular tolerance of approximately ±2° at the −20 dB criterion, while Figure 5c shows the joint $\left(\Delta \psi ,\Delta \theta \right)$ response. The approximately ±6° phase and ±2° angle intervals are one-variable cuts for the Table 1 parameters and sequences, not universal MRTCM tolerance bounds. For multi-pulse validation, the narrowband configuration uses B=10 MHz, $T_{p}=8\;\mu s$, PRT=60 μs, and a 128-pulse coherent processing interval (CPI); other parameters remain as specified in Table 1.

**Figure 6:**
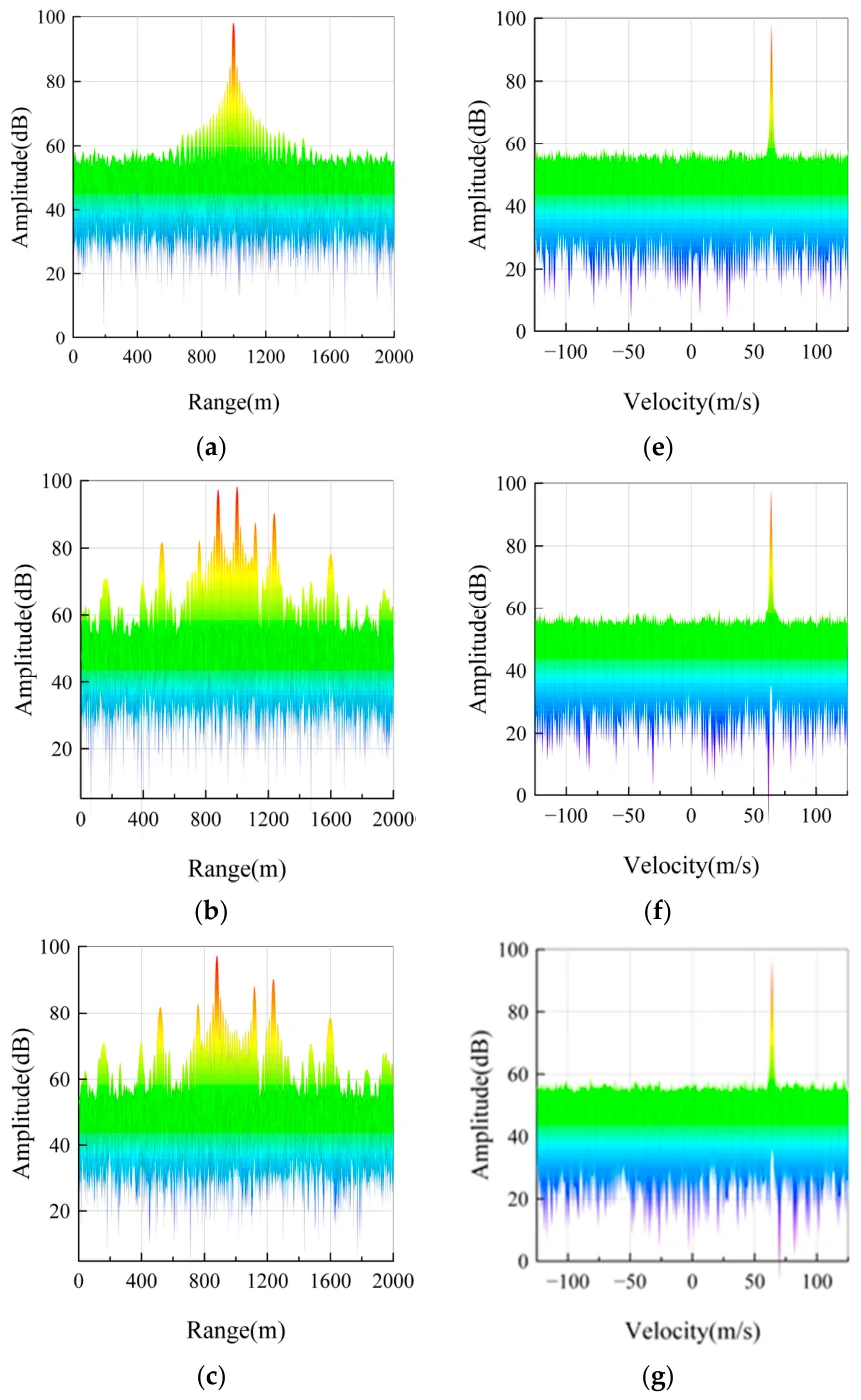

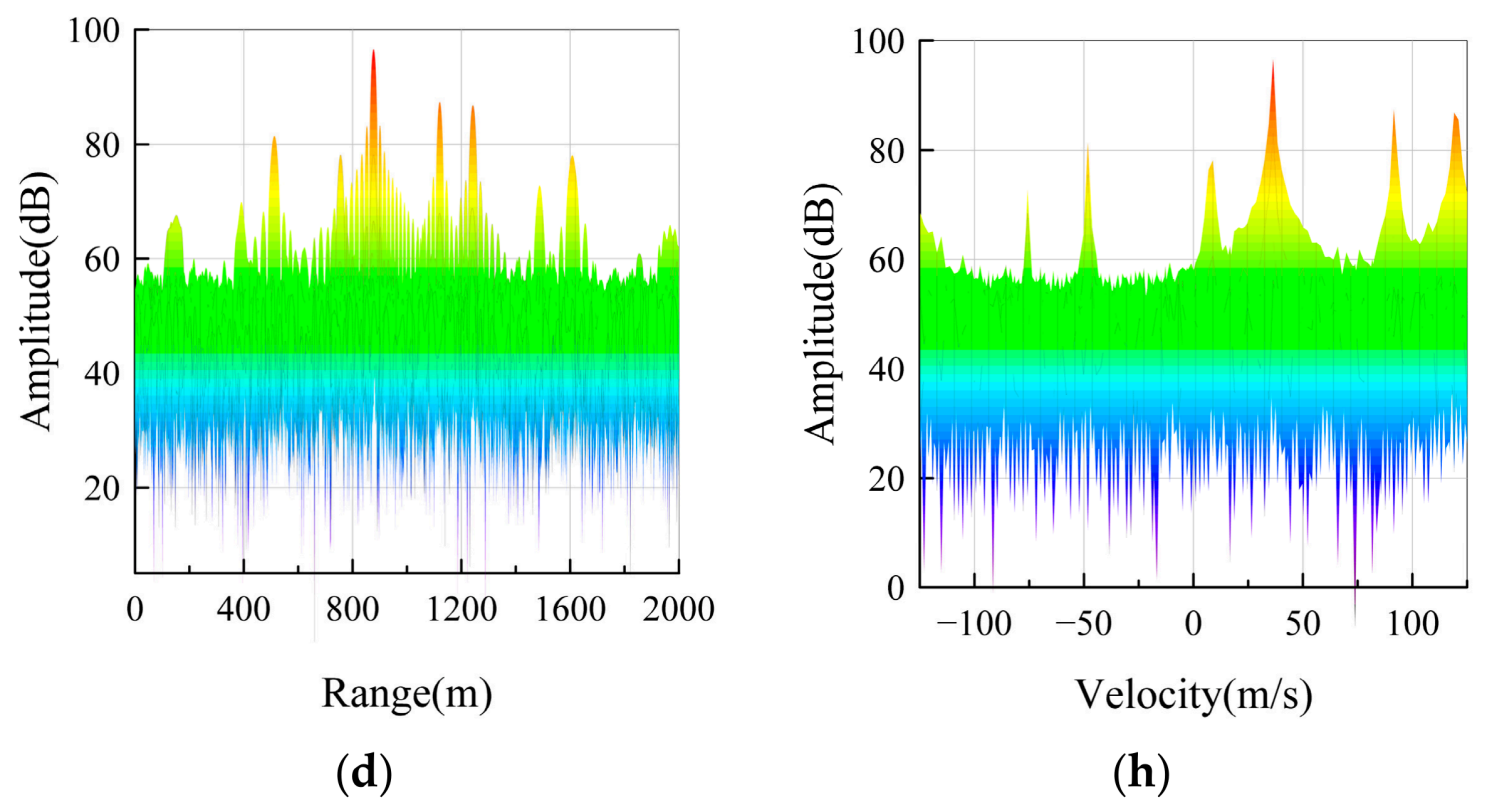
Moving-target detection (MTD) results for zeroth-order-component-based cancellation under 128-pulse coherent integration. (**a**) Target range profile. (**b**) Modulated metasurface with f_s_ = 1 MHz. (**c**) Synchronized cancellation result. (**d**) Asynchronous result with f_s_ = 1.0069 MHz. (**e**) Target Doppler response. (**f**) Modulated metasurface Doppler response. (**g**) Synchronized cancellation in Doppler. (**h**) Asynchronous cancellation with Doppler shift. Different colors are used only to distinguish the individual pulse responses.

Figure 6 compares moving-target detection (MTD) results. The target at $R_{1}=1000$ m has radial velocity $v_{1}=62.5$ m/s. For $f_{s}=1$ MHz and PRT=60 μs, $f_{s}/PRF=PRT/T_{m}=60\in Z$. The zeroth-order contribution is matched at the target range sample, whereas higher-order components remain at other ranges and may alias in Doppler. The synthesized response suppresses the target component by more than 20 dB across the displayed Doppler channels.

Figure 6d,h use $f_{s}=1.0069$ MHz, so successive pulses sample different coding phases. The zeroth-order cancellation vector is unchanged, whereas the higher-order phases and Doppler aliases may vary. These results verify code-start invariance of the zeroth-order component; the higher-order spectrum remains code-phase dependent.

More generally, if the modulation frequency is expressed as $f_{s}=PRF\left(N+p/q\right)$, where p/q is irreducible, the sampled modulation phase repeats every q radar pulses. This periodic structure can be exploited to control the higher-order Doppler aliases without affecting the zeroth-order cancellation; the resulting alias pattern depends on q and on the coding sequence.

### 4.2. Harmonic Component Performance in HRRP and MTD Processing

Beyond co-range cancellation, the MRTCM system enables range-separated target manipulation through controllable harmonic generation. The u-th-order harmonic appears at an apparent range offset $\Delta R_{u}=-u\cdot c\cdot f_{s}/\left(2K_{r}\right)$ from the metasurface physical location, facilitating both false target generation and range-separated target cancellation.

Table 2 summarizes the system parameters for harmonic-based range-separated cancellation. The target (region 1) remains positioned at $R_{1}$ = 1000 m, while the time-coding metasurface regions (regions 2 and 3) are located at $R_{2}$ = $R_{3}$ = 985 m, establishing a 15 m physical separation in the range dimension. The element spacing along the y-direction remains $d_{y}$ = 15 mm, with observation angle θ = 37°. The modulation frequency $f_{s}$ = 0.5 MHz is selected to position the −1st-order harmonic precisely at 1000 m, coinciding with the target location.

**Figure 7:**
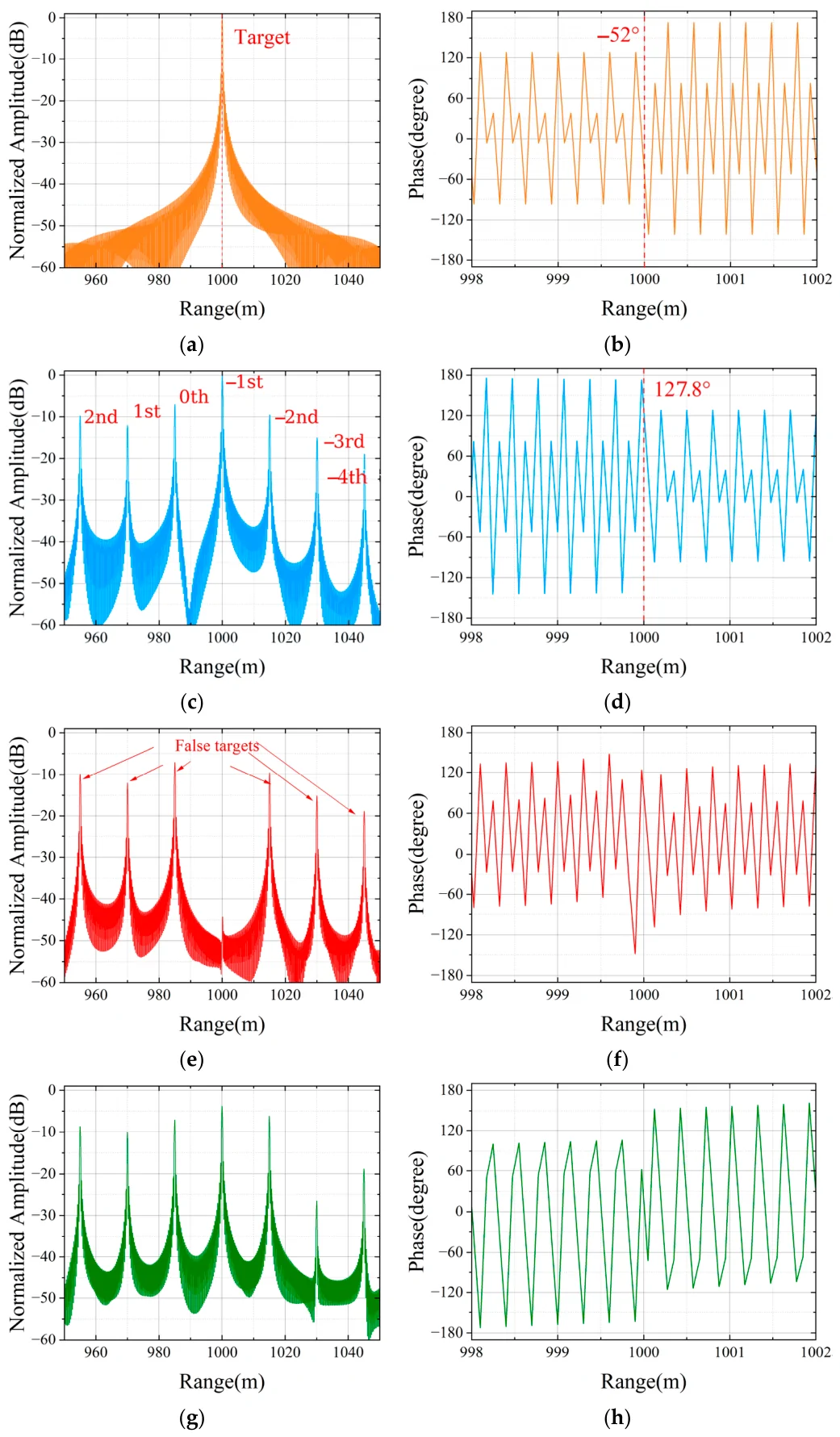
Complex HRRP of harmonic-based range-separated cancellation with 15 m range separation. (**a**) Target amplitude at 1000 m. (**b**) Target phase. (**c**) Modulated metasurface amplitude at 985 m showing the u = −1 harmonic at the target location. (**d**) Modulated metasurface phase. (**e**) Synthesized amplitude demonstrating 48.3 dB suppression. (**f**) Synthesized phase. (**g**) Amplitude after independently applying a one-chip cyclic registration shift to each controller, showing cancellation failure. (**h**) Phase after the cyclic shift.

Figure 7 presents harmonic-based range-separated cancellation using the same target-range phase reference as Figure 4. Although regions 2 and 3 are physically located at 985 m, Equation (13) retains their actual delays and harmonic peak phases. At the 1000 m target sample, their u=−1 components sum to approximately 1.00227∠127.845°. The relative phase with the 1∠(−52°) target is 179.845°, producing a deep null consistent with the sampled −48.3 dB response. As in Figure 4f, the phase at the deep null in Figure 7f is ill-conditioned.

For u≠0, a cyclic shift preserves coefficient magnitude but rotates its phase. Figure 7g,h apply a one-chip shift to each regional controller separately. Because the two sequences have different chip durations, the cases represent independent controller-registration errors rather than a common propagation delay. In each case the selected u=−1 phase is disturbed and the target response reappears.

For multi-pulse radar scenarios, harmonic-based range-separated cancellation requires both initial pulse-arrival/code-phase knowledge and harmonic recurrence, $uf_{s}/PRF\in Z$. To evaluate these constraints, a modified narrowband radar configuration is employed. The target is positioned at 1000 m, whereas the two coded regions are located at 850 m. The waveform and modulation parameters are selected so that the u=−1 harmonic undergoes a 150 m apparent range shift and coincides with the target range cell. The regional sequences are [2, 2, 1, 3] and [1, 1, 1, 1, 1, 3, 3, 3].

**Figure 8:**
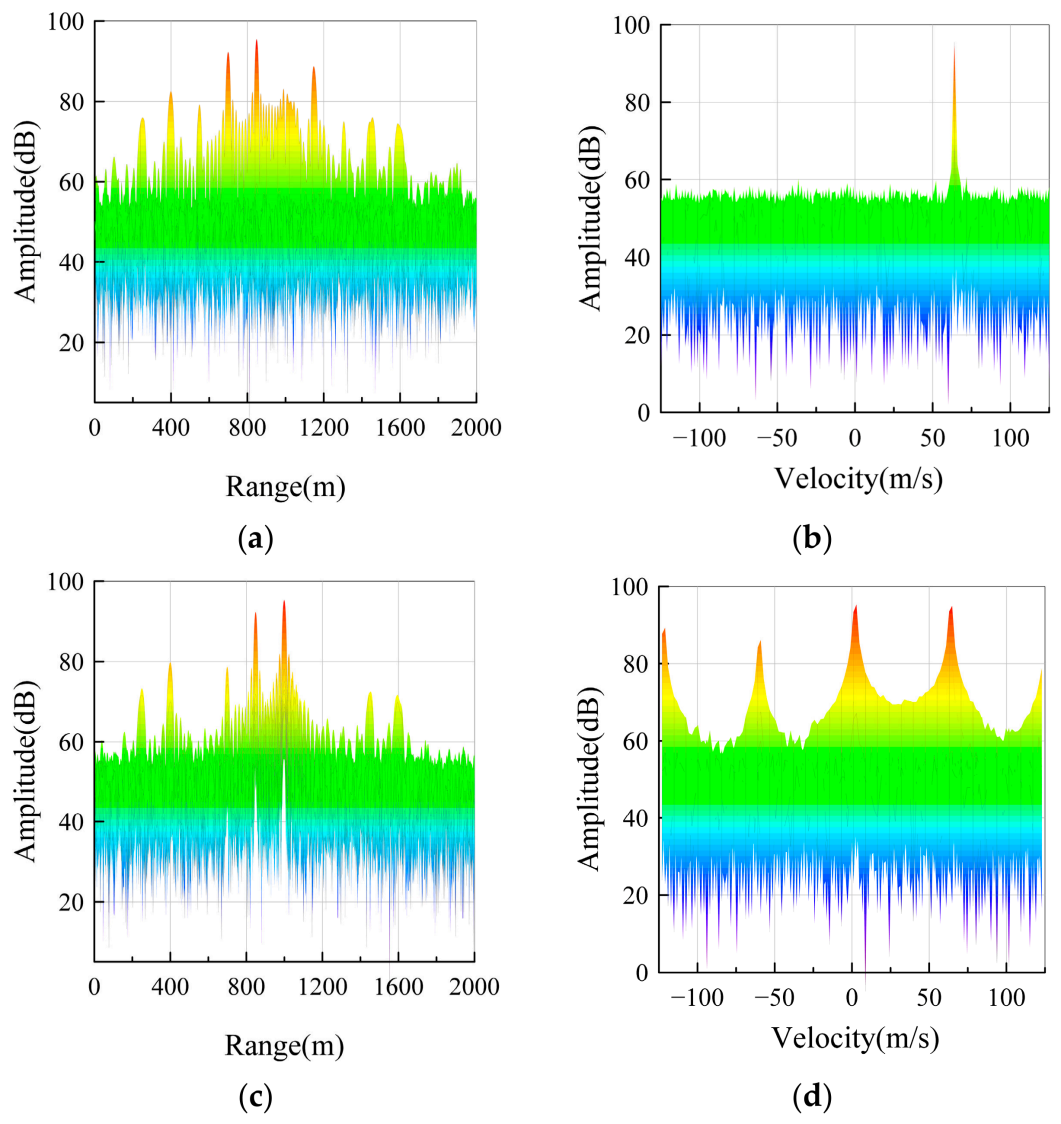
MTD results for harmonic-based range-separated cancellation under a 128-pulse coherent processing interval. (**a**) Range profile under synchronized modulation. (**b**) Corresponding Doppler response, showing range-domain suppression with a residual Doppler signature. (**c**) Range profile under asynchronous modulation. (**d**) Corresponding Doppler response, showing degradation of cancellation in both the range and Doppler dimensions. Different colors are used only to distinguish the individual pulse responses.

Figure 8 presents synchronized and asynchronous MTD results. In the synchronized case, $uf_{s}/PRF\in Z$ fixes the coding-induced harmonic phase from pulse to pulse and enables range-sample suppression. The remaining Doppler signature shows that range alignment and code-phase recurrence alone do not reproduce the target slow-time phase.

In the asynchronous case, $uf_{s}/PRF$ is noninteger, so the selected harmonic rotates from pulse to pulse. Stable complex matching is lost and the target response persists in both range and Doppler.

These results demonstrate that the operational effectiveness of harmonic-based range-separated cancellation is strongly constrained by timing knowledge and radar–metasurface synchronization.

## 5. Conclusions

This paper presents a multi-region time-coding metasurface (MRTCM) architecture for electromagnetic protection of platforms where comprehensive metasurface coverage is impractical. By coordinating distributed metasurface regions to manipulate scattered fields from uncovered areas, the proposed method supports target concealment together with harmonic false target generation.

Theoretical analysis establishes the cyclic-shift invariance of the zeroth-order Fourier coefficient $\alpha_{0}$, and the numerical results confirm its value for asynchronous co-range cancellation. The simulations demonstrate more than 40 dB target suppression in high-resolution range profiles and effective cancellation under moving-target detection processing with coherent pulse integration. By contrast, range-separated cancellation using higher-order harmonics requires accurate time-of-arrival information and synchronization.

The simulations use an uncoupled far-field point-scatterer model, with frequency- and angle-independent coefficients over the simulated interval. The reported cancellation depths and tolerance ranges therefore apply to the stated numerical cases rather than to measured hardware. Future experimental work will require coupling-aware regional calibration and full-wave modeling of finite-array effects, including edge treatment [26].

## Figures and Tables

**Table 1:** Co-range cancellation parameters.

Object/Region	Symbol	Physical Meaning	Value
Radar	$f_{c}$	Carrier frequency	10 GHz
Radar	B	linear frequency-modulated (LFM) bandwidth	500 MHz
Radar	$T_{p}$	Pulse duration	100 μs
Target	$\sigma_{1}$	Effective field-amplitude coefficient	1
Target	$\psi_{1}$	Intrinsic scattering phase	68°
Target	$R_{1}$	Target range	1000 m
Geometry	θ	Observation angle	37°
Geometry	$d_{y}$	Region-center spacing	15 mm
Geometry	$R_{2}=R_{3}$	Cancellation-region ranges	1000 m
MRTCM region 2	$\sigma_{2}$	Field-amplitude coefficient	1
MRTCM region 2	$f_{s}$	Modulation frequency	1 MHz
MRTCM region 2	Sequence	2-bit state-index sequence	[2, 3, 3]
MRTCM region 3	$\sigma_{3}$	Field-amplitude coefficient	1
MRTCM region 3	$f_{s}$	Modulation frequency	1 MHz
MRTCM region 3	Sequence	2-bit state-index sequence	[1, 2, 4]

**Table 2:** Range-separated cancellation parameters.

Object/Region	Symbol	Physical Meaning	Value
Radar	$f_{c}$	Carrier frequency	10 GHz
Radar	B	LFM bandwidth	500 MHz
Radar	$T_{p}$	Pulse duration	100 μs
Target	$\sigma_{1}$	Effective field-amplitude coefficient	1
Target	$\psi_{1}$	Intrinsic scattering phase	68°
Target	$R_{1}$	Target range	1000 m
Geometry	θ	Observation angle	37°
Geometry	$d_{y}$	Region-center spacing	15 mm
Geometry	$R_{2}=R_{3}$	Cancellation-region ranges	985 m
MRTCM region 2	$\sigma_{2}$	Field-amplitude coefficient	1
MRTCM region 2	$f_{s}$	Modulation frequency	0.5 MHz
MRTCM region 2	Sequence	2-bit state-index sequence	[1, 1, 1, 3, 3, 2, 2, 1]
MRTCM region 3	$\sigma_{3}$	Field-amplitude coefficient	1
MRTCM region 3	$f_{s}$	Modulation frequency	0.5 MHz
MRTCM region 3	Sequence	2-bit state-index sequence	[1, 4, 2, 4, 2, 2, 2, 2, 2, 2, 1, 1, 3, 1, 2, 1, 2, 1]

## Data Availability

The data presented in this study are available from the corresponding authors upon reasonable request.
